# A case of monosomy 21 presented with difficult tracheal intubation

**DOI:** 10.1186/s40981-022-00511-w

**Published:** 2022-03-25

**Authors:** Yoshiki Saito, Tomohiro Chaki, Noriaki Nishihara, Michiaki Yamakage

**Affiliations:** Department of Anesthesiology, Sapporo Medical University School of Medicine, 291, South 1, West 16, Chuo-ku, Sapporo, Hokkaido Japan

**Keywords:** Chromosomal disorders, Congenital abnormalities, Airway management

## Abstract

**Background:**

Monosomy 21 is a rare chromosomal abnormality. It is mainly associated with mental retardation, intellectual disability, growth retardation, microcephaly, and characteristic facial features. General anesthesia in adults with this disease has not been reported. We report difficult airway management of an adult patient with monosomy 21.

**Case description:**

A 30-year-old female was scheduled for laparoscopic gynecological surgery. She was diagnosed with monosomy 21 at birth and accompanied with mental retardation. Preoperative examination revealed limited mouth opening with Mallampati score of IV, but no abnormal laboratory test or chest X-P. Anesthesia was performed using general anesthesia with epidural analgesia. Although bag-mask ventilation was improved by a muscle relaxant, mouth opening was further restricted, and laryngoscope insertion was impossible. Tracheal intubation was achieved using a bronchofiberscope. The operation procedure was completed, and the patient was discharged from the hospital without any major postoperative complications.

**Conclusions:**

In this patient, mouth opening was further reduced after induction of general anesthesia with a muscle relaxant. Preoperative evaluation and adequate preparation of airway management are important for general anesthesia in an adult patient with monosomy 21.

## Background

Monosomy 21 is a rare chromosomal abnormality because most of the fetuses with complete monosomy 21 die before birth. The severity of symptoms varies depending on the size and localization of the defect of chromosome 21 [1, 2]. Reported symptoms include growth retardation, ear abnormalities, seizures, microcephaly, protruding occiput, abnormal muscle tone, and cardiac and brain diseases [2, 3]. General anesthesia in a toddler has been reported, but there have been few reports on adult cases of monosomy 21 and anesthetic management for such patients [4]. In the case of that toddler, no difficult ventilation or intubation was reported during repeated general anesthesia with halothane. In this article, we report difficult airway management in an adult patient with monosomy 21.

## Case presentation

We obtained written informed consent from the patient and one of her parents for publication of this case report. The manuscript adheres to the CAse REport (CARE) guidelines.

The patient was a 30-year-old woman, with a height of 140 cm and weight of 40 kg. She was born with a low birth weight. A chromosomal test was performed after birth, and she was diagnosed with 21 monosomy, although the details of chromosomal defect are unclear. She had a history of hospitalization for pneumonia as an infant but had not had any illness requiring hospitalization since then, and there was no family history. Her intellectual level was equivalent to a 5-year-old child due to mental retardation. She was scheduled for total laparoscopic hysterectomy and bilateral salpingo-oophorectomy for a uterine cervical tumor. Preoperative airway assessment revealed that cervical retroflection was normal, but the interincisal distance was 3 cm, the mandible was retracted and difficult to protrude, and the Mallampati score was class IV, and airway assessment using the modified Arné score showed a simplified score of 21, indicating that tracheal intubation would be difficult [5]. Mallampati score class IV and mandibular advancement difficulties were risks of mask ventilation difficult. Preoperative examinations including blood test, electrocardiogram, chest X-ray, and transthoracic echocardiography showed no significant findings. The patient had exercise tolerance of more than 4 metabolic equivalents.

General anesthesia with epidural anesthesia was planned. We considered that general anesthesia with total intravenous anesthesia using propofol should be performed because of the possibility of skeletal muscle disease since abnormal muscle tone has been reported as one of the symptoms of monosomy 21 [6]. In addition, tracheal intubation with McGRATH™ MAC (Covidien, Dublin, Ireland) after general anesthesia induction was planned for airway management.

The patient entered the operating room calmly without any problems. After entering the room, standard American Society of Anesthesiologists monitors including a percutaneous oxygen saturation monitor, electrocardiogram, and noninvasive blood pressure monitor were attached, and a venous catheter was inserted. Administration of oxygen at 6 L/min was then started, and a bispectral index monitor was placed on the patient’s forehead. Muscle relaxation monitoring was performed using TOF watch® (Organon Ireland Limited, Organon, Ireland). An electrode was attached to the ulnar side of the patient’s left forearm to monitor the ulnar nerve-maternal adductor muscle. We started target-controlled infusion of propofol at an effect site concentration of 5.0 μg/ml in addition to remifentanil 0.15 μg/kg/min. Train-of-four (TOF) monitoring was performed when the bispectral index fell below 60 and a TOF count of 4/4 was confirmed. Mandibular elevation was difficult, and the interincisal distance was about one fingerbreadth. Moreover, her oral cavity was almost completely occupied by the tongue. Since mask ventilation was difficult, 30 mg (0.75 mg/kg) of rocuronium was immediately administered. Mask ventilation became easier with a tidal volume of approximately 200 ml after administration of rocuronium. Since insertion of a McGRATH™ MAC blade size 3 was impossible because of the restriction of mouth opening, tracheal intubation using a bronchial fiber was performed with caregiver elevation of the mandible. Oxygenation was maintained during the intubation procedure. An epidural catheter was inserted at the Th12/L1 level. We decreased the target concentration of propofol to 2.7 μg/ml, while infusion rate of remifentanil was unchanged during surgery for maintaining BIS between 40 and 60. A TOF count of 4/4 was confirmed about 50 min after the first dose of muscle relaxant, and muscle relaxation was maintained with 10 mg rocuronium administered every 30 mins.

No major problems occurred in anesthesia management during the surgery. The anesthesia time was 4 h and 5 min, and the operation time was 2 h and 5 min. After completion of the operation, remifentanil infusion was discontinued, and 80 mg of sugammadex was intravenously administered. Propofol infusion was discontinued after confirming adequate tidal volume (more than 300 ml) under 5 cmH_2_O of pressure support and 4 cmH_2_O of positive end expiratory pressure. The patient was then extubated when body movements were observed. After extubation, no abnormalities were observed in respiratory and circulatory states. The patient was transferred to the hospital ward. Postoperatively, no anesthesia-related complications were observed, and the patient was discharged 7 days after surgery.

## Discussion

We reported difficult airway management of an adult patient with monosomy 21. There were two problems in this case. The first was the predicted difficulty in securing the airway, and the second was the possibility that the patient had skeletal muscle disease.

In the present case, the modified Arné score was used for preoperative airway assessment [7]. The Wilson score is also considered to be useful for predicting intubation difficulty [8]. Both scores include assessment of mandibular protrusion by the upper lip bite test. This is a very useful finding alone to predict difficulty in intubation [9]. Our patient had difficulty in mandibular protrusion and had Mallampati score class IV, predicting difficulty in intubation. In cases of difficulty in mouth opening, tracheal intubation procedures may be limited. Bronchial fibers and tracheal tube introducers may be useful. However, tracheal intubation should be performed under direct vision using a bronchial fiber if anatomical abnormalities of the head and neck are suspected.

A supraglottic airway (SGA) device is effective for difficult ventilation and difficult intubation. Monosomy 21 can cause various symptoms depending on the site of chromosomal deletion. In our patient, in addition to oral cavity volume problems and mandibular abnormalities, there may have been temporomandibular joint abnormality. Insertion of an SGA device may be effective in a patient with severe airway obstruction during induction of anesthesia. Mask ventilation and intubation are very difficult in patients with temporomandibular joint abnormalities. According to past reports, insertion of an SGA device is an effective procedure for securing the airway in such cases [10]. However, it was thought that insertion of an SGA device would not be effective in our patient because the patient’s mouth opening was severely restricted, and tracheal intubation with a bronchial fiber was considered to be the most effective procedure.

Monosomy 21 has also been reported in patients with abnormal muscle tone [6]. Therefore, although there had been no diagnosis of skeletal muscle disease, its presence should be assumed in our patient. In patients with neuromuscular diseases such as myasthenia gravis and muscular dystrophy, careful management of muscle relaxation should be performed under muscle relaxation monitoring. Our patient did not show excessive muscle relaxation as in patients with the abovementioned diseases, but it took 7 min for the TOF count to be 0/4 even with 0.75 mg/kg of rocuronium administration. This is a large deviation from the reported time of onset of the effect of rocuronium in Japanese, and it may indicate some abnormality in the sensitivity to a muscle relaxant [11]. Our patient did not receive any anticonvulsant or antiepileptic medications preoperatively, and there was no other reason found in medications to diminish the effect of muscle relaxants [12, 13]. Adult patients with monosomy 21 may have reduced sensitivity to muscle relaxants.

## Conclusion

Monosomy 21 is a rare congenital anomaly. Difficulty in securing the airway in our patient was preoperatively expected due to anatomical abnormalities, but restricted mouth opening was deteriorated after induction of anesthesia. Symptoms of monosomy 21 vary depending on the site of chromosomal deletion, and careful preoperative examination of each patient is therefore necessary for safe anesthetic management.

## Data Availability

Data sharing is not applicable to this article as no datasets were generated or analyzed during the current study.

## Abbreviations

TOF: Train-of-four
SGA: Supraglottic airway
